# Effect of polydeoxyribonucleotide injection on pes anserine bursitis: A case report

**DOI:** 10.1097/MD.0000000000008330

**Published:** 2017-10-27

**Authors:** Jong-Uk Mun, Hyung R. Cho, Sae M. Bae, Soo K. Park, Soo .l Choi, Mi S. Seo, Young S. Lim, Soo H. Woo RN, Young U. Kim

**Affiliations:** a Department of Orthopedic Surgery, Changwon Gyeongsang National University Hospital, Incheon; b Department of Anesthesiology and Pain Medicine, Myongji Hospital, College of Medicine, Seonam University, Goyang; c Department of Anesthesiology and Pain Medicine, Dongtan Sacred Heart Hospital, Hallym University College of Medicine, Incheon; d Department of Anesthesiology and Pain Medicine, Catholic Kwandong University of Korea College of Medicine, International ST. Mary‘s Hospital, Incheon; e Department of Nursing, Kyung-In Women‘s University, Incheon, Republic of Korea.

**Keywords:** case report, glucocorticoid, pes anserinus bursitis, polydeoxyribonucleotide

## Abstract

**Rationale:**

Pes anserine (PA) bursitis is an inflammatory condition of the medial knee. The PA bursa becomes more painful when infected, damaged, or irritated. Although various treatment options have been attempted to treat PA bursitis, optimal treatments are still debated. This study aims to investigate the effect of polydeoxyribonucleotide (PDRN) injection on reducing pain and inflammation in a patient presenting with PA bursitis.

**Patient concerns:**

A 50-year-old female patient was admitted to our pain clinic with symptoms of tenderness and pain over the medial knee. Physical examination revealed the pain to be located over the proximal medial tibia at the insertion of the conjoined tendons of the PA. The knee had lost its range of movement and strength, and resisted knee flexion.

**Diagnoses:**

She was diagnosed as having PA bursitis.

**Interventions:**

Ultrasound guided PA bursa injection was carried out.

**Outcomes:**

Follow-up for the patient was more than eight months. She showed good improvement in PA bursitis without any complications.

**Lessons:**

This is the first successful report of successful PDRN injection for PA bursa.

## 1 Introduction

Pes anserine (PA) bursitis is a common soft tissue pain disease affecting the medial knee.^[1,2]^ The gracilis, sartorius, and semitendinosus tendons along the proximal part of the tibia constitute the PA.^[3]^ The bursa is located beneath the PA. Most patients recover spontaneously, by activity modification and conservative management.^[4]^ However, PA bursitis occasionally results in intractable pain and requires long-term therapy for recovery.^[4]^ It can also trigger weakness of hip adductors and weakness of medial hamstring muscles.^[4]^ Ultrasound facilitates the diagnosis of PA bursitis.^[5]^ Initial treatment includes nonsteroidal anti-inflammatory drugs (NSAIDs) or ice to reduce inflammation and pain.^[4]^ Rehabilitation of these patients includes increasing the stretching, flexibility, and endurance of PA muscles.^[4]^ Use of short-term knee orthosis may be suitable for reducing the pain.^[6,7]^ In cases when intractable pain causes functional limitations or where conservative managements fail, local glucocorticoid injection is administered.^[4]^ However, glucocorticoid use is limited as it may cause multiple adverse effects such as, glucocorticoid-induced osteoporosis,^[8,9]^ and mood and cognitive disorders.^[8]^ Polydeoxyribonuclotide (PDRN, Placentexingergro; Mastellisrl, San Remo, Italy) is derived from *Oncorhynchus mykiss* (Salmon trout) or *O. keta* (Chum Salmon), and is noted as substitute for glucocorticods.^[10]^ PDRN has anti-inflammatory effects, lowering the expression of the inflammatory cytokines such as tumor necrosis factor-alpha and interleukin-6. Moreover, previous studies demonstrated that PDRN has no adverse effects.^[10]^ We report here a patient with PA bursitis who underwent ultrasound guided PA bursa PDRN injection for management of intractable knee pain.

## 2 Case presentation

**Figure 1:**
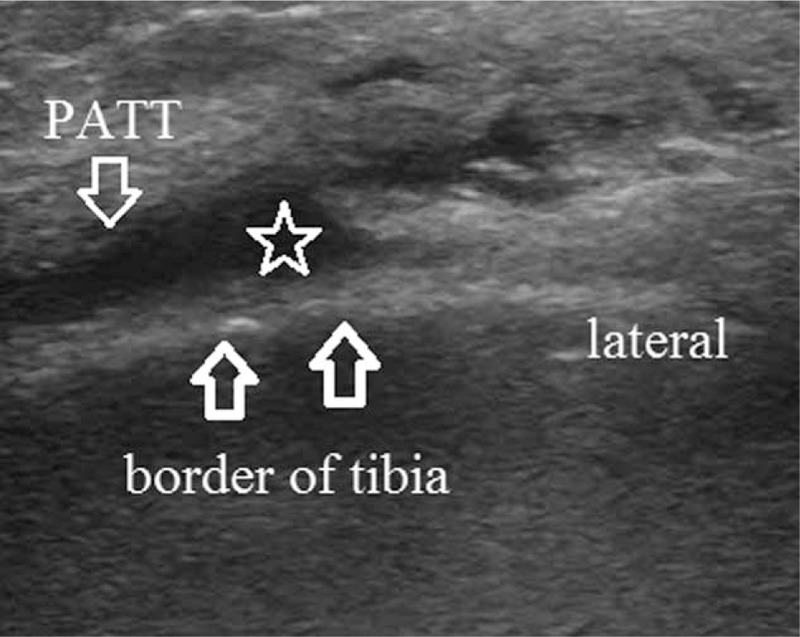
Ultrasound image showing increased fluid collection within the pes anserine bursa (asterix). PATT = pes anserine tendon thickening.

**Figure 2:**
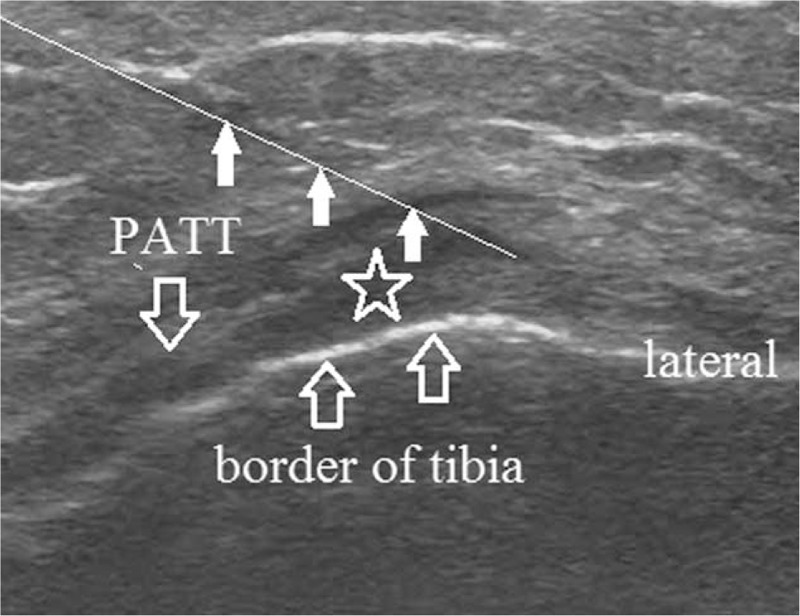
Ultrasound image during injection of polydeoxyribonucleotide in the pes anserine bursa (asterix). White arrow indicates the block needle. PATT = pes anserine tendon thickening.

A 50-year-old female patient was admitted to our pain clinic with symptoms of tenderness and pain over the knee, with local swelling over the medial aspect of the left knee. The patient reported chronic refractory pain in the inner knee area during aggravating daily activities, which exacerbated when she used the stairs. At the time of admission, the patient was unable to walk because of severe pain, rated 7/10 on the Numeric Rating Scale (NRS). Physical examination revealed pain over the proximal medial tibia at the insertion of the conjoined tendons of the PA. The knee had lost its range of movement and strength, and resisted knee flexion. She complained of pain when she stretched her hamstrings. She stood 149.5 cm tall, weighing 51.6 kg, with body mass index of 23.09 kg/m^2^. She had a past history of hypothyroidism, cervical disc herniation, and golfer‘s elbow. She was receiving physical therapy, such as compression bandages and NSAIDs for intractable pain of the medial knee, but showed no improvement. Ultrasound imaging revealed increased fluid collection within the bursa, thickened bursa wall, and surrounding tissue edema (Fig. 1). The patient declined to use glucocorticoid bursa injection after the effects and side effects, such as glucocorticoid-induced osteoporosis, adrenal suppression, and cognitive and mood disorder, were explained to her. We then presented to her the anti-inflammatory effects of PDRN. Verbal and written informed consent was obtained from the patient before the procedure. The patient was brought to the ultrasound room and placed on the table in a supine position with bent knees. We performed ultrasound guided PA bursa injection using 5.625 mg/3 mL of PDRN, 1% lidocaine 1.5 mL with a 26-G, 4-cm needle (Fig. 2). At the 1 week follow-up after the PA bursa PDRN injection, the NRS score had decreased from 7 to 2. However, the patient reported a persistent pain in her left knee. No adverse reactions were observed. At the 2 weeks follow-up, the patient reported significant pain reduction with decreased NRS scores from 2 to 0. Follow-up was continued for more than eight months, and the patient has recovered completely. She had no pain and had a full range of motion of the left knee during walking. We also investigated the side effects of PDRN injection during follow-up. Fortunately, no side-effects have been noted.

## 3 Discussion

We present the case of a female patient with PA bursitis, who successfully received PA bursa PDRN injection for left medial knee pain.PA bursitis is common causes of knee pain leading to pain in the anteromedial area of the knee.^[4]^ Classic symptoms are swelling and tenderness over the medial knee, or medial proximal tibia pain mimicking medial meniscal tear,^[11]^ or medial collateral ligament.^[2,3]^ Repetitive rotator movement on the knee, trauma, contusion, and excessive valgus, can cause tendinitis and bursitis due to friction on the PA bursa.^[2]^ The presence of PA bursitis also increases the severity of walking disability in knee osteoarthritis.^[2]^ Different types of bursitis, including prepatellar, popliteal, and PA bursitis, are the causes of knee pain of patients referring to pain clinic.^[4]^ Treatment includes physiotherapy, NSAIDs, and injections of glucocorticoid with highly variable responses.^[12]^ However, a number of treatments only provide a short-term benefit, or may not be satisfactorily effective.^[13]^ Moreover, some procedures are associated with risks. For example, although glucocorticoid injections provide temporary pain reduction, care should be taken to avoid injecting any of the 3 tendons converging at the PA. Injections within the tendons can intensify the pain and further weaken these structures.^[4]^ Extracorporeal shock wave therapy (ESWT) is now spotlighted as an alternative treatment for PA bursitis. ESWT increases the neovascularization, along with increased angiogenic growth index in the tendon, bone, and tendon-bone junction.^[4]^ However, ESWT also has unwanted side effects such as transitory reddening of the skin, pain, and small hematomas.^[14]^

Conversely, PDRN, whose action is derived from anti-inflammatory effects that promote wound healing by tissue regeneration, has no side effects.^[15,16]^ PDRN repairs and regenerates the cellular damage by interacting with A2 purinergic receptor and stimulating the production of vascular endothelial growth factors.^[15]^ PDRN had recently found a wide application in the medical field. Lim et al^[15]^ reported the effectiveness of PDRN injection in posterior tibial tendon dysfunction patients undergoing ankle syndesmotic surgery. Won-Joong et al^[17]^ demonstrated the effectiveness of PDRN injection in ischiofemoral impingement syndrome patients who were not indicated for surgery. Chronic and acute toxicity studies confirm that PDRN has no toxic effects on the brain, lungs, heart, liver, and skeletal muscle on macroscopic and histologic analysis.^[15]^

Based on the safety and the efficacy of PDRN presented in previous studies, we decided to administer PDRN and received informed consent from the patient, after giving a detailed explanation of the procedure. We conducted PA bursa PDRN injection. According to the results of the current study, PA bursa PDRN injection could be an effective method for treating and reducing PA bursitis pain without any adverse effects. This is the first successful report on ultrasound guided PA bursa injection of PDRN. Further studies on the efficacy and safety of PDRN are required, dealing with the diverse use of PDRN. In addition, we hope you to expect our upcoming randomized controlled clinical trial on PA bursa PDRN injection. The take-away message from this case report is that PDRN injection for PA bursa is an efficient and safe therapeutic option for the treatment of PA bursitis.
